# Enriched Bone Marrow Derived Disseminated Neuroblastoma Cells Can Be a Reliable Source for Gene Expression Studies—A Validation Study

**DOI:** 10.1371/journal.pone.0137995

**Published:** 2015-09-11

**Authors:** Fikret Rifatbegovic, M. Reza Abbasi, Sabine Taschner-Mandl, Maximilian Kauer, Andreas Weinhäusel, Rupert Handgretinger, Peter F. Ambros

**Affiliations:** 1 CCRI, Children’s Cancer Research Institute, St. Anna Kinderkrebsforschung, Vienna, Austria; 2 Molecular Diagnostics, Health & Environment Department, AIT Austrian Institute of Technology GmbH, Vienna, Austria; 3 Children's University Hospital, University of Tuebingen, Tuebingen, Germany; 4 Department of Pediatrics, Medical University of Vienna, Vienna, Austria; University of Navarra, SPAIN

## Abstract

**Background:**

Metastases in the bone marrow (BM) in form of disseminated tumor cells (DTCs) are frequent events at diagnosis and also at relapse in high-risk neuroblastoma patients. The frequently highly diluted occurrence of DTCs requires adequate enrichment strategies to enable their detailed characterization. However, to avoid methodical artifacts we tested whether pre-analytical processing steps—including transport duration, temperature and, importantly, tumor cell enrichment techniques—are confounding factors for gene expression analysis in DTCs.

**Methods:**

LAN-1 neuroblastoma cells were spiked into tumor free BM and/or peripheral blood and: i) kept at room temperature or at 4°C for 24, 48 and 72 hours; ii) frozen down at -80°C and thawed; iii) enriched via magnetic beads. The effect on the gene expression signature of LAN-1 cells was analyzed by qPCR arrays and gene expression microarrays.

**Results:**

Neither storage at –80°C in DMSO and subsequent thawing nor enrichment of spiked-in neuroblastoma cells changed the expression of the analyzed genes significantly. Whereas storage at 4°C altered the expression of analyzed genes (14.3%) only at the 72h-timepoint in comparison to the 0h-timepoint, storage at room temperature had a much more profound effect on gene expression by affecting 20% at 24h, 26% at 48h and 43% at 72h of the analyzed genes.

**Conclusion:**

Using neuroblastoma as a model, we show that tumor cell enrichment by magnetic bead separation has virtually no effect on gene expression in DTCs. However, transport time and temperature can influence the expression profile remarkably. Thus, the expression profile of routinely collected BM samples can be analyzed without concern as long as the transport conditions are monitored.

## Introduction

With the development of new genomic and transcriptomic techniques over the past two decades, our understanding of molecular mechanisms in various tumors has increased dramatically. Gene expression profiling by microarray analysis and other technologies has enabled the classification of a number of adult and pediatric tumors, as well as the prediction of patient outcomes [1–5]. Some of these gene expression signatures have been implemented in FDA approved tests which are already commercially available and are used to identify patients who benefit from specific treatment protocols [6].

In neuroblastoma (NB), which is the most common extracranial cancer of early childhood [7, 8], several studies presented gene expression signatures of primary tumors that predict patient outcome [1, 9–14]. Despite the fact that bone marrow (BM), as a special source for liquid biopsy [15], is by far more accessible than tumor samples (BM aspirates are routinely obtained at different time points in current high risk neuroblastoma studies, e.g. HR-NBL1) and that it is a common organ for disseminated tumor cells (DTCs) in NB [16], the expression profile of enriched DTCs was studied so far from one group [17]. However, the results concerning differences in the expression profile between NB patients being alive vs dead were only of minor impact. Thus, further well-designed studies are required to better uncover the expression differences among differently behaving patients. In contrast, in leukemia research BM samples are already used as a source for gene expression profiling studies [18–21].

Virtually all biospecimens experience a number of manipulations prior to analysis. Depending on the goal of the analysis and the targets to be analyzed, these pre-analytical manipulations can influence the results of the study. Whereas DNA remains usually stable during the various pre-analytical manipulations and sample storage [22, 23], RNA is more prone to degradation due to its biochemical instability and sensitivity to RNases. In addition, RNA transcription is highly regulated and can be influenced by different factors [24]. Surprisingly, results of various studies concerning the impact of pre-analytical manipulations on RNA integrity and gene expression are not consistent. Several studies analyzing the impact of warm ischemia prior to sample processing on RNA integrity showed that the RNA is stable up to 24h at room temperature [25–28], whereas other studies showed that RNA is less stable under the same conditions [29–32]. In addition, several studies analyzed the impact of various pre-analytical manipulations on the gene expression signatures of biospecimens, showing that gene expression alterations occur at different rates, probably depending on the tissue type [26, 33–36]. Neuroblastoma cells present the GD2 ganglioside on the cell membrane at high concentration making this molecule an ideal marker to quantify neuroblastoma cells [37] but also to enrich these cells via magnetic bead-based technology. In our recent studies we have shown that the enriched disseminated NB cells from BM samples can serve as an excellent source of high quality DNA for molecular diagnostics by ultra-high density SNParrays [38, 39]. However, the impact of pre-analytical handling, i.e. transport of the samples at room temperature or at 4°C, storage at -80°C and magnetic bead-based enrichment of disseminated NB cells on their gene expression signature, has not been addressed so far.

## Material and Methods

### Cell culture

The human NB cell line LAN-1, kindly provided by Dr. R Seeger, USA, was grown in RPMI 1640 medium (Life Technologies) supplemented with 10% fetal bovine serum (PAA), 2.5% HEPES buffer (PAA), 1% sodium pyruvate solution (PAA) and 0.7% penicillin/streptomycin (PAA). Cells were grown to 60–80% confluence before spiking and RNA extractions.

### Collection of peripheral blood and BM samples

Peripheral blood (PB) of healthy volunteers was collected in heparin coated blood collection tubes (Vacuette). Tumor-free BM aliquots were obtained from leftover material of samples obtained for diagnostic procedures within the course of a clinical study. Written consent was given by the patient/parent to do the diagnostic test and to do research on left over material. The study ‘Analyse von Tumorzellexpressionsmarkern von Neuroblastompatienten mit disseminiertem Stadium’ was approved by the St. Anna Kinderspital Ethics Commission on the 9^th^ July 2014 in Vienna, Austria.

### Spiking and enrichment of LAN-1 cells

1x10^6^ LAN-1 cells were spiked into fresh PB and tumor-free BM. The PB and BM were diluted 1:1 with cold RPMI media (Life Technologies) and mononuclear cells (MNCs) were isolated by density gradient separation (Lymphoprep, Axis Shield) according to standard methodology. Enrichment of LAN-1 from the MNC fraction was performed by magnetically activated cell sorting (MACS, Miltenyi) applying FITC labeled anti-GD2 antibody (delta CH2 clone) and anti-FITC microbeads as described earlier [38]. Both, density gradient centrifugation and magnetic bead-based separation were performed at 4°C. Small aliquots of the enriched fraction were used for cytospin preparation and GD2-immunofluorescence/DAPI staining. The enrichment efficiency was estimated by counting GD2^POS^ cells by fluorescence microscopy (Zeiss) as described earlier [38].

### Freezing and thawing of MNCs

Isolated MNCs, including spiked-in tumor cells, were centrifuged for 10 minutes at 1300 rpm and 4°C. The pellet was resuspended in RPMI 1640 medium (Life Technologies) and diluted 1:1 with DMSO (Serva) to a final volume of 1.8 ml with 20% DMSO. The cells were frozen at -80°C by using cell freezing containers (Biocision) that allow controlled freezing (-1°C per minute).

After storage at -80°C for seven days, the samples were thawed by transferring the whole cell solution to 10 ml of ice-cold RPMI 1640 medium (Life Technologies). The cells were centrifuged for 10 minutes at 1300 rpm and 4°C, and the supernatant was discarded. Finally, the pellet was resuspended in MACS buffer (PBS, Life Technologies; 0.5 mM BSA, Sigma Aldrich; 2 mM EDTA, Life Technologies).

### qPCR analysis

Total RNA was extracted by TRIzol (Life Technologies) according to the manufacturer´s protocol. Quantity and quality of RNA were determined by the ND-1000 spectrophotometer (Thermo Scientific) and RNA integrity by Experion (BioRad) according to the manufacturer´s instructions. For qPCR analysis, 200 ng of total RNA was reverse-transcribed into cDNA using an oligo dT 20 primer (VBC biotech), Reaction Buffer (Promega), 4x10 mM dNTP Mix (Promega), 40 U/μl Recombinant RNasin Ribonuclease Inhibitor (Promega), 200 U/μl M-MLV Reverse Transcriptase (Promega) and RNase free H2O (Qiagen). Reverse transcription was performed in a T3000 thermocycler (Biometra). Annealing was performed for 5 minutes at 70°C and 5 minutes at 4°C. The reverse transcription reaction was done with the following program: 60 minutes at 42°C and 15 minutes at 70°C. The qPCR analysis was done with the 96.96 Dynamic Array IFC (Fluidigm) using a precast palette of 96 cancer oriented genes according Fluidigm’s recommendations. In short cDNA were preamplified for 22 cycles with all the 96 primer pairs at a concentration of 200nM of each primer in a standard PCR setup in 10μl volumes. Then pre-amplicons were diluted 1:5 with water loaded into 96x96 microfluidic qPCR arrays together with each of 96 single primer PCR mixes including Eva Green for detection using Fluidigm’s HX-IFC Controller (Fluidigm). PCR primers and specific conditions are available on request. Cp-values were extracted from qPCR runs with the Biomark-software (Fluidigm) and used for further bioinformatic analyses.

### Microarray Analysis

For GeneChip PrimeView Human Gene Expression analysis (Affymetrix), total RNA was extracted by TRIzol (Life Technologies) and treated with RQ1 RNase–Free DNase (Promega) according to the manufacturer´s protocol. Prior to microarray analysis, total RNA was purified according to the RNeasy MinElute Cleanup (Qiagen) protocol. 100ng total RNA was used for the GeneChip PrimeView Human Gene Expression analysis following the manufacturer´s protocol. The *in vitro* transcription was performed for 16 hours, and the hybridization was done in the GeneChip Hybridization Oven 640 (Affymetrix). The GeneChip Scanner 3000 was used for scanning.

### Bioinformatic Analysis

CEL files from Affymetrix Primeview arrays were further processed in R statistical environment using Bioconductor packages [40]. Summarized log_2_ probe set signals were calculated using RMA [41]. Subsequently the most variable (across all samples) probe set was chosen for each gene for further analysis. Differential gene expression between groups of samples (N = 3 biological replicates for each different treatment of cells) was determined using the limma package [42]. P values for differential expression analysis were adjusted for multiple testing by the Benjamini-Hochberg method [43].

Further analyses of qPCR data were performed in R statistical environment using Bioconductor packages [40]. For each gene qPCR-CT values of technical replicas on the same 96 well array were averaged and subsequently these values were normalized to the mean of 3 control genes (*ALDOA*, *RPL11*, *RPL19*). For each treatment 3 biological replicates were done and differential gene expression values between the different treatment options were determined using the limma package [42]. P values for differential expression analyses were adjusted for multiple testing by the Benjamini-Hochberg method [43]. Genes were omitted if they had failed results in any of the conditions.

## Results and Discussion

### Incubation of spiked-in tumor cells at 4°C and at room temperature influences the expression profile of tested genes moderately and strongly, respectively.

**Fig 1:**
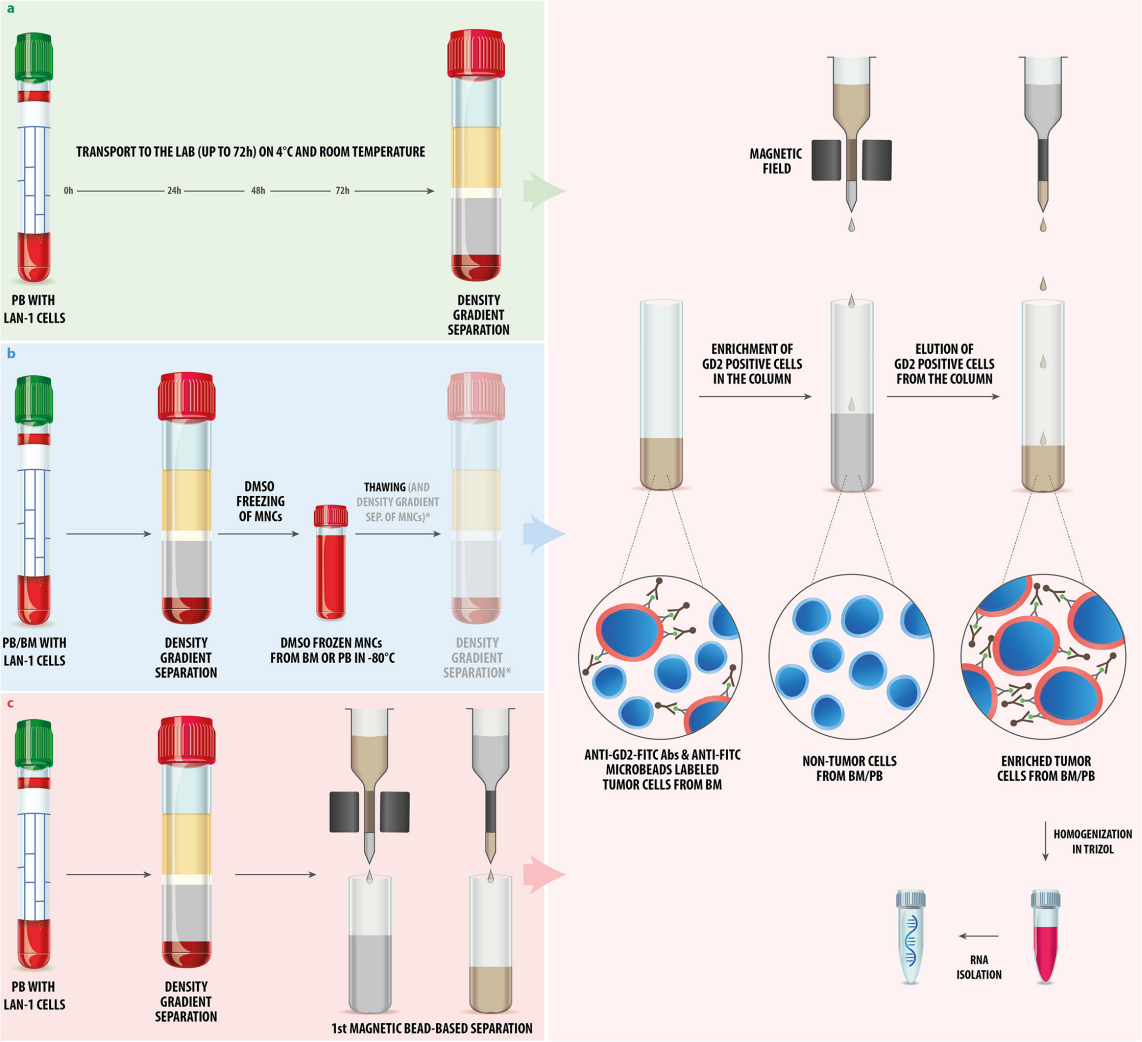
Experimental design. **(a)** We spiked LAN-1 NB cells into fresh PB and kept the samples for 0, 24, 48 and 72h at room temperature and, for the same time periods, at 4°C prior to density gradient separation. The LAN-1 cells were enriched from the MNC fraction with magnetic beads to a 99% purity of the tumor cell fractions prior to homogenization in TRIzol. RNA was isolated from all seven samples simultaneously and used for the qPCR array. (**b)** LAN-1 cells were spiked into PB and tumor-free BM, and density gradient separation was immediately performed. The MNCs were frozen in 20% DMSO for seven days at -80°C. After thawing, the LAN-1 cells were either directly enriched by magnetic bead-based separation, or an additional density gradient separation (*) was performed prior to magnetic bead-based separation. The samples were homogenized in TRIzol and the isolated RNA was used for qPCR (in case of PB) and microarrays (in case of BM). **(c)** LAN-1 cells were spiked into PB and density gradient separation of MNCs was performed without delay, following two enrichment steps in a row. The >99% LAN-1 cell fractions were homogenized in TRIzol and RNA was isolated from all samples simultaneously. qPCR arrays were performed in order to analyze the effect of enrichment on selected genes.

We addressed the impact of three pre-analytical handling steps on the gene expression signature of spiked-in NB cells: i) storage (= transport to the lab), ii) freezing/thawing and storage at -80°C, and iii) density gradient separation and magnetic bead-based enrichment of NB cells. An overview of all pre-analytical handling procedures is shown in Fig 1.

**Fig 2:**
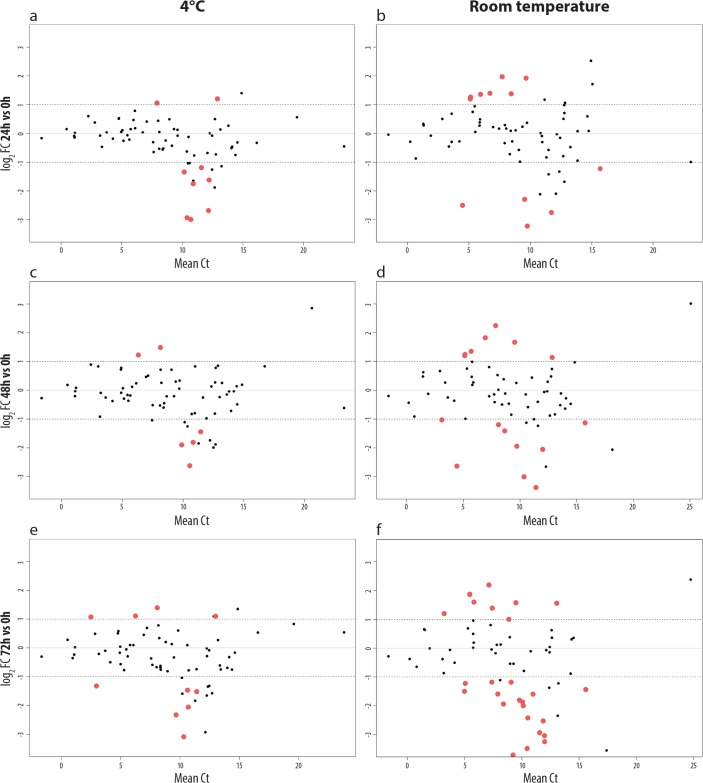
Altered gene expression of NB cells kept at 4°C and at RT up to 72h. 70 genes were analyzed by qPCR array. The altered gene expression of LAN-1 cells kept for 24 hours in PB on 4°C (a) and at room temperature (b) is shown. In (c) and (d) the same is shown for 48h, and in (e) and (f) for 72h. Red dots represent genes that are significantly changed (p<0.05, |log_2_FC|>1) at given time points compared to the baseline (time point 0h). Genes with |log_2_FC|>1 and labeled by black dots, did not show significant changes (p>0.05) between the three biological replicates. The log_2_ fold change for a given conditions is indicated on the y-axis, whereas the mean Ct value is shown on the x-axis.

To address the question of how the gene expression of tumor cells in PB changes during transportation time (= incubation time), we analyzed 70 genes in spiked-in tumor cells incubated for 0, 24, 48 and 72 h at 4°C and for the same time periods at room temperature by the qPCR 96 well array (Fig 1). Fresh PB was aliquoted in seven 2 ml fractions. One aliquot, serving as baseline for the analysis (0h), was immediately processed (density gradient separation > magnetic bead-based enrichment > TRIzol homogenization), while the other six samples were first incubated at 4°C or at room temperature for 24h (n = 2), 48h (n = 2) and 72h (n = 2) (Fig 2).

At 4°C the expression of nine analyzed genes (12.87%) was significantly changed (p<0.05, |log_2_FC|>1) at 24 h, six genes (8.57%) at 48 h and ten genes (14.29%) after 72 h. Even more gene expression alterations—as compared to the immediately processed sample—were observed when PB samples were stored at room temperature, which resulted in a significant change (p<0.05, |log_2_FC|>1) of 14 genes (20%) after 24 h, 18 genes (25.71%) after 48 h and 30 genes (42.86%) after 72 h (Fig 2).

At 4°C, the expression of only four genes (5.71%) was found to be significantly altered at all three time points (*MAP1B*, *ITGAL*, *MUC5AC* and *CRLF1*). *MAP1B* encodes a microtubule associated protein that is essential for neuritogenesis [44], and it was the only down-regulated gene during all three time points at 4°C. MAP1B mRNA expression was found to be abundantly expressed in proliferating and differentiating cells of the developing central nervous system [45]. The MAP1B protein is also known to be associated with p53 in neuroblastoma cell lines decreasing its activity and inhibiting doxorubicin-induced apoptosis [46]. *ITGAL*, one of the three up-regulated genes, encodes a cell adhesion protein. In a former study, it was shown that the surface expression of the protein could be increased by hyperthermia in a neuroblastoma cell line [47]. The gene expression of MUC5AC was found to be altered by temperature increase in a non-neuroblastoma model [48], whereas no considerable studies associated with CRLF1 gene expression and neuroblastoma or temperature dependence are present. As some genes we found altered in our study are of interest for the neuroblastoma research community, it is important to consider that alterations of these genes seen in other studies can be, at least in part, a consequence of sample storage.

At room temperature, 11 genes (15.71%) had an altered gene expression level at all three time points (*RXRA*, *VEGFA*, *RXRB*, *CA4*, *BMP7*, *CXCL1*, *PDGFA*, *COMP*, *GRM8*, *CDKN2C*, and *CYP27A1*), which is a significant increase compared to the storage at 4°C where we found only four genes altered at all three time points. An overview of all altered genes is shown in the S1 Table.

The two at room temperature up-regulated genes, *VEGFA* and *PDGFA*, encode growth factors that have been found to be significantly higher expressed in advanced neuroblastomas as compared to low stage tumors, with *PDGFA* being associated with patient survival [49]. Authors have also suggested anti–VEGFA treatment for high-risk neuroblastoma patients [50]. However, data concerning VEGFA expression in neuroblastoma are not consistent [51], which could be, at least in part, due to different storage conditions of the biological samples.

Notably, all samples in our study had RQI values ranging from 7.9–10.0, and no trend in RNA degradation during incubation was observed (S2 Table). These observations exclude the possibility that the gene expression alterations were mainly due to RNA degradation, but are rather due to transcriptional regulation. However, the alterations at room temperature could also be, at least in part, a consequence of tumor cells being attacked by NK cells, macrophages or T-cells in the peripheral blood of the unrelated donor (personal communication S. Asgharzadeh).

Our results show that the gene expression signature of NB cells is altered considerably during time delays at room temperature prior to analysis or storage at -80°C in DMSO. This is in agreement with similar findings [26], emphasizing that, ideally, no shipment of fresh BM should be undertaken, and in case no other possibility exists, shipping to the lab should be done at low temperature and, ideally, be monitored. According to published studies, there is a tissue-dependent effect of sample storage at RT. In colon tissue the first significant gene expression alteration was observed already 15 minutes after surgery [52], whereas in breast tissue the expression was stable for two hours at room temperature [27]. In case of circulating tumor cells in peripheral blood of breast cancer patients, first significant alterations of gene expression were observed already after 4 h, while the expression of other genes was stable for 48 h [53]. As our data suggest, this effect can be moderated by keeping the samples at 4°C instead of keeping them at room temperature. To obtain highly reliable data from gene expression studies, BM samples should be processed immediately or frozen within few hours after aspiration.

### Freezing and thawing of spiked tumor cells does not systematically alter their expression signature

We evaluated the impact of freezing, storage at -80°C and thawing of PB/BM samples on the gene expression of spiked-in NB cells. LAN-1 cells were spiked into three PB and three BM samples. All samples were used for density gradient separation. In the first experimental set up, the BM and PB samples were enriched for GD2^pos^ LAN-1 cells and homogenized in TRIzol immediately after spiking, whereas two other BM and PB samples were frozen in 20% DMSO and stored for seven days at -80°C in DMSO. After thawing, the second set of PB and BM samples were enriched for GD2^pos^ cells, followed by homogenization in TRIzol. The third group of PB and BM samples, after one additional density gradient centrifugation step, underwent the same further steps as the samples described above (Fig 1).

**Fig 3:**
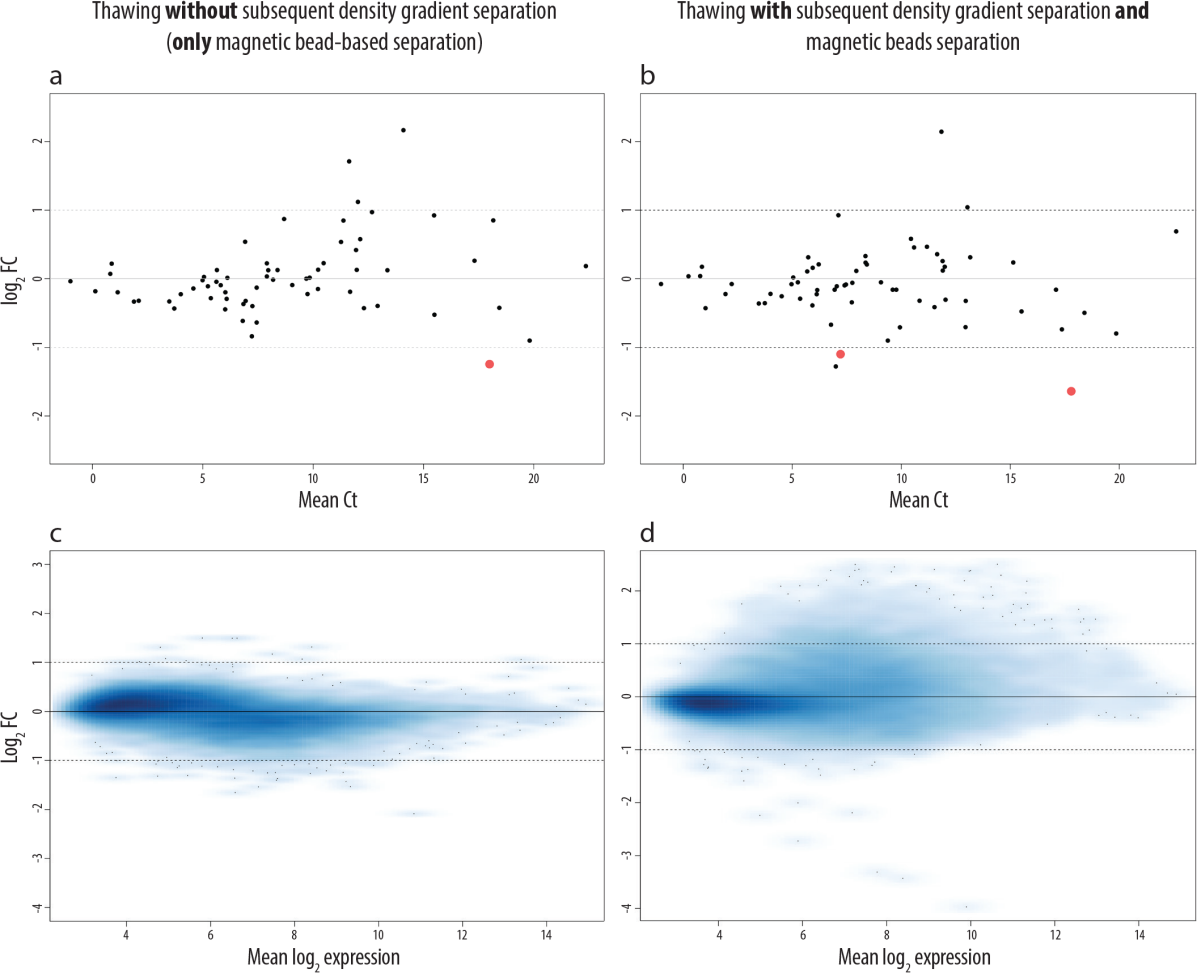
Effects of freezing, storage and thawing of samples on NB gene expression. In (a-b) we analyzed the effect of freezing, storage at -80°C and thawing by qPCR array on the expression of 64 genes of NB cells spiked into PB. In (a) we show the effect when after thawing the samples were only enriched by magnetic bead-based separation, whereas in (b) we introduced an additional density gradient separation prior to magnetic bead-based separation. Red dots represent genes that are significantly changed (p<0.05, |log_2_FC|>1) at given conditions compared to the baseline (LAN-1 cells enriched and homogenized in TRIzol immediately after spiking). Genes with |log_2_FC|>1 and p>0.05 are represented by black dots (not significant), as their expression was not coherently changed between the three biological replicates. The log_2_ fold change is indicated on the y-axis and the mean Ct values in the x-axis. In (c-d) we analyzed the same effects on NB cells spiked into BM by microarrays. The mean log_2_ expression is shown in the x-axis, and the log_2_ fold change on the y-axis.

After RNA isolation, we performed a qPCR analysis of 64 genes on the spiked tumor cells in PB, and microarray analyses of 19960 genes on tumor cells spiked in BM (Fig 3). The qPCR data indicate that the expression of one gene was significantly changed (p<0.05, |log_2_FC|>1) if, after thawing of PB, no additional density gradient separation step was introduced (Fig 3A). The expression of two genes (3.13%) was significantly changed (p<0.05, |log_2_FC|>1) when an additional density gradient separation step was performed (Fig 3B). One gene (*CDKN2C*) was altered in both post-thawing procedures. However, no overall trend in gene expression changes could be observed in the analyzed genes and replicates (S3 Table).

The microarray analysis of 19960 genes from spiked-in and recovered LAN-1 cells revealed that freezing, storage and thawing without the additional density gradient separation altered only 22 genes (0.15%) significantly (p<0.05, |log_2_FC|>1). However, when an additional density gradient separation step was introduced after thawing, the number of significantly altered genes (p<0.05, |log_2_FC|>1) increased to 505 genes (2.53%).

**Fig 4:**
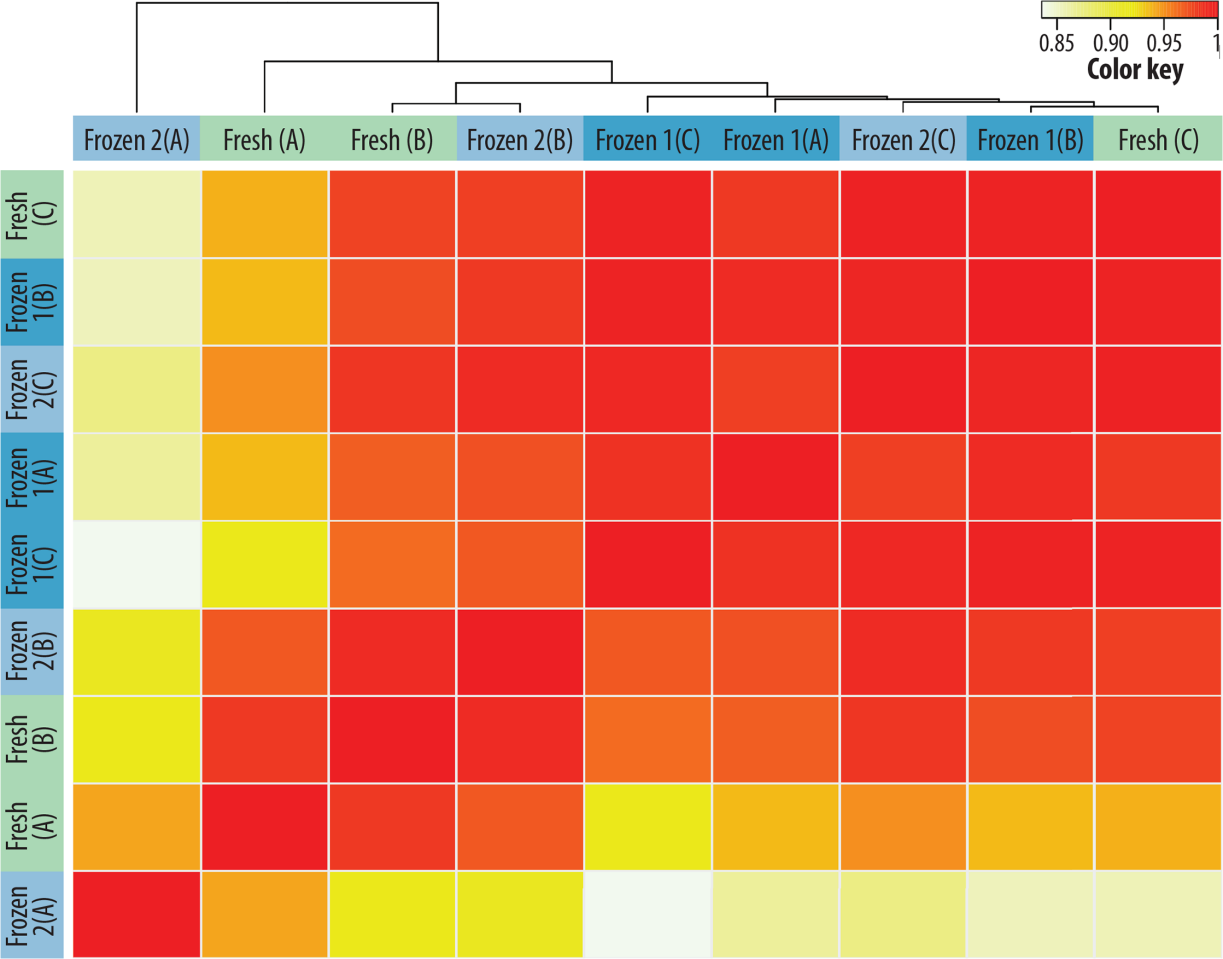
Unsupervised clustering of NB samples after freezing, storage and thawing procedure. Microarray analysis of three biological replicates (A-C) and the three different pretreatment conditions: fresh (no freezing/thawing), frozen 1 (thawing > magnetic bead-based separation of LAN-1 cells) and frozen 2 (thawing > density gradient separation > magnetic bead-based separation of LAN-1 cells). In the unsupervised clustering of the expression of the analyzed genes, the fresh and the two differently frozen samples (1, 2) did not cluster. The correlation coefficient (R) is illustrated by the color key: white (0) = no correlation and red (1) = high correlation.

In the unsupervised clustering of the microarray data, the fresh and frozen samples of the three biological replicates did not cluster (Fig 4), indicating that the freezing/thawing procedure and storage at -80°C does not systematically affect the gene expression pattern of spiked-in NB cells.

### Magnetic bead-based enrichment of NB cells has no significant impact on their gene expression

**Fig 5:**
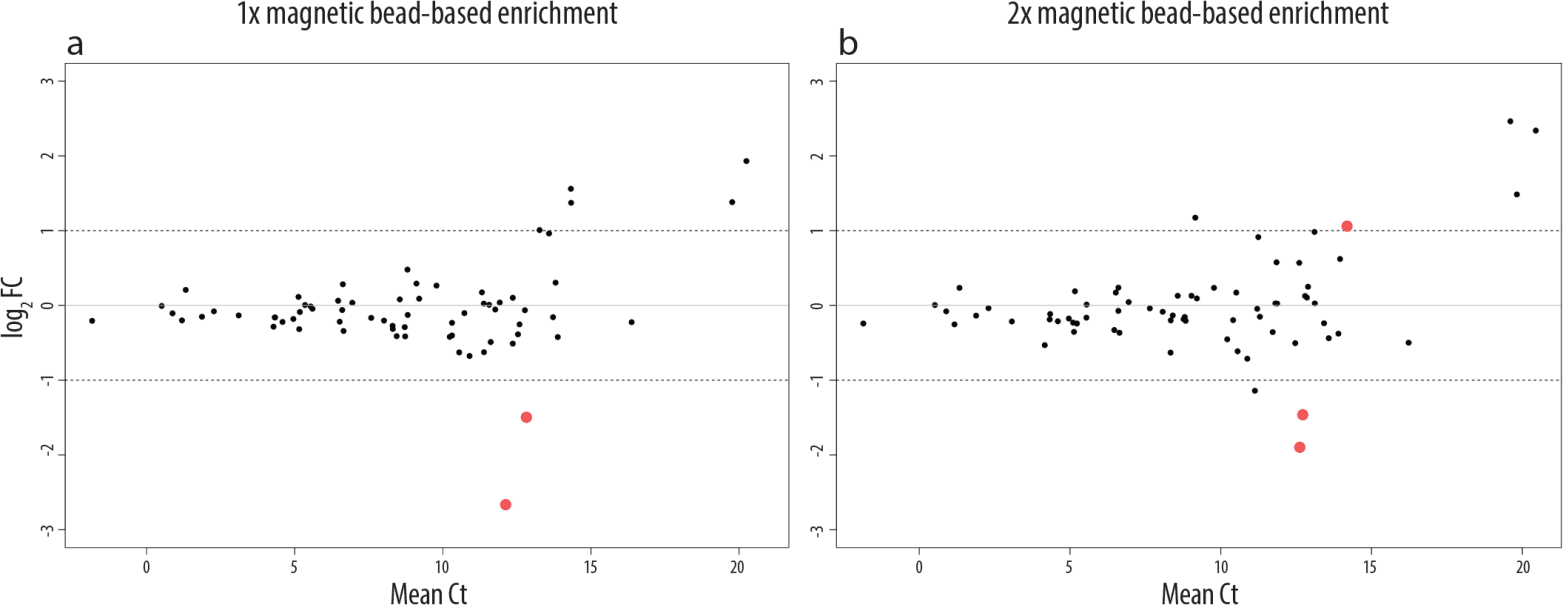
Effects of magnetic bead-based enrichment of NB cells on their gene expression. qPCR arrays were used to analyze the effects of magnetic bead-based enrichment on the expression of 71 genes in NB cells. In (a) the altered gene expression is shown for cells that have been enriched only once after density gradient separation, whereas in (b) the effect of two following magnetic bead-based enrichment steps is shown. Red dots represent genes that are significantly changed (p<0.05, |log_2_FC|>1) at given conditions compared to the baseline (LAN-1 cells before spiking into PB). The expression of genes with |log_2_FC|>1 but p>0.05 are not considered as significant, as their expression was not coherently changed in the different biological replicates. The log_2_ fold change is indicated on the y-axis and the mean Ct values in the x-axis.

To assess the issue of whether magnetic bead-based enrichment of DTCs affects the expression profiles of tumor cells, we performed qPCR analysis of 71 genes on spiked-in LAN-1 cells. For this purpose we spiked LAN-1 cells into PB and performed the density gradient separation without delay. Following density gradient separation, LAN-1 cells were enriched twice in a row. We analyzed the impact of a repeated magnetic bead-based enrichment, as in some cases a one-step magnetic bead-based enrichment does not result in the requested purity of the DTCs. The gene expression profiles of LAN-1 cells were analyzed after each enrichment step and compared to the gene expression signature of non-manipulated LAN-1 cells (cells that have not been spiked into PB, but have been homogenized in TRIzol). Independently of the number of enrichments, only three genes (4.23%) were significantly altered (p<0.05, |log_2_FC|>1, Fig 5). These results indicate that density gradient separation alters the gene expression of spiked-in tumor cells slightly more than magnetic bead-based enrichment, which, in contrast, has virtually no effect on the gene expression of neuroblastoma cells under conditions explained above (S4 Table).

The impact of immunomagnetic bead-based enrichment on the gene expression signature was so far mainly studied on peripheral blood cells [54, 55]. These studies support our findings that the impact of magnetic bead-based enrichment on the gene expression is negligible. However, these studies examined different antibody–receptor combinations and so far no data are available concerning a short treatment of neuroblastoma cells with anti GD2 antibodies at 4°C, although an anti-neuroblastoma effect is known in *in vivo* and *in vitro* systems when the antibody is applied on neuroblastoma cells for longer periods [56]. Of all pre-analytical handling steps performed in this study the magnetic bead-based enrichment with GD2 antibodies has the least impact on the gene expression signatures of neuroblastoma cells.

## Conclusion

Our results show that of all pre-analytical handling steps, the resting-time (corresponding to the transport time) at room temperature, prior to analysis or storage at -80°C, altered the gene expression of spiked-in NB cells the most. The gene expression alterations could, however, be reduced if the samples were kept at 4°C. Taken together, reliable expression results of DTCs can be obtained if the transport of the BM sample does not take longer than 24 h at 4°C. However, in order to obtain unbiased results, immediate DTC enrichment and RNA extraction, or freezing of the complete BM in DMSO for future separation, should always be preferred.

Further, we were able to show that freezing, thawing and storage at -80°C did not systematically alter the gene expression of spiked-in NB cells. And, finally, magnetic bead-based enrichment of DTCs had no marked effect on the expression profile of the spiked-in tumor cells. Thus, this technique is an excellent tool to enrich DTCs suitable for gene expression analysis.

## Supporting Information

S1 Table Influence of incubation at 4°C and room temperature (= transport) of PB samples on the expression signature of spiked-in NB cells. The file contains the log2 fold changes, p values and adjusted p values for multiple testing by the Benjamini-Hochberg method (q values) of all analyzed genes.
(XLS)

S2 Table RQI values of samples incubated for up to 72h. RQI values listen in an excel file (.xls). Samples were stored at room temperature and 4°C. The experiment was repeated three times.
(XLS)

S3 Table Influence of freezing/thawing and storage at -80°C of PB samples on the gene expression signature of spiked-in NB cells. The file contains the log_2_ fold changes, p values and adjusted p values for multiple testing by the Benjamini-Hochberg method (q values) of all analyzed genes.
(XLS)

S4 Table Influence of density gradient separation and magnetic bead-based enrichment of NB cells on their gene expression. The file contains the log_2_ fold changes, p values and adjusted p values for multiple testing by the Benjamini-Hochberg method (q values) of all analyzed genes.
(XLS)
